# Determinants of mental health distress among health workers in Abu Dhabi, United Arab Emirates

**DOI:** 10.3389/fpsyt.2026.1756380

**Published:** 2026-06-02

**Authors:** Emad Masuadi, Yasir Ahmed Mohammed Elhadi, Aminu S. Abdullahi, Souheila AliHassan, Iffat Elbarazi

**Affiliations:** 1 Institute of Public Health, College of Medicine and Health Sciences, United Arab Emirates University, Al-Ain, United Arab Emirates; 2 Education Department, Abu Dhabi Health Services Company, Abu Dhabi, United Arab Emirates

**Keywords:** DASS-21, health workforce, mental health, occupational health, UAE

## Abstract

Healthcare workers (HCWs) remain at elevated risk of psychological distress, yet post-pandemic evidence from the United Arab Emirates is scarce. This cross-sectional electronic survey assessed depression, anxiety, and stress among physicians and nurses providing direct clinical care across Abu Dhabi Health Services Company in March 2024. Mental health outcomes were measured using the DASS-21, and multivariable logistic regression identified associated factors. Among 383 HCWs (mean age 40.8 years; 70% female; 65% nurses), the prevalence of depression, anxiety, and stress was 49%, 45%, and 46%, respectively. Middle Eastern HCWs had significantly higher odds of depression (Adjusted Odds Ratio (aOR)=1.94; 95% CI: 1.13–3.35), anxiety (aOR=1.80; 95% CI: 1.03–3.21), and stress (aOR=2.42; 95% CI: 1.37–4.33) compared with Asian HCWs. Workplace conflict strongly predicted distress as conflicts with colleagues increased the odds of depression (aOR=3.36; 95% CI: 1.92–6.05), anxiety (aOR=1.99; 95% CI: 1.14–3.53), and stress (aOR=3.78; 95% CI: 2.10–7.00). Chronic fatigue was associated with depression (aOR=4.21; 95% CI: 1.61–13.2), anxiety (aOR=3.89; 95% CI: 1.39–13.8), and stress (aOR=3.34; 95% CI: 1.32–9.63). Protective factors included age ≥40 years for stress (aOR=0.61; 95% CI: 0.39–0.96) and postgraduate education for anxiety (aOR=0.55; 95% CI: 0.31–0.94) with nurses had lower stress than physicians (aOR=0.47; 95% CI: 0.23–0.94). Post-pandemic mental-health symptoms remain substantial among Abu Dhabi HCWs, particularly younger providers and those exposed to workplace conflict or chronic fatigue. Sustained mental-health programs, routine screening, and targeted support for high-risk groups are urgently required to protect clinician well-being and healthcare system resilience.

## 1 Introduction

Healthcare workers (HCWs) experience a consistently higher burden of mental health problems than the general population (1). During the COVID−19 pandemic, international estimates suggested that around one in four HCWs reported symptoms of depression, and substantial proportions reported anxiety and stress (2). Chronic exposure to high workloads, long shifts, moral distress, and patient suffering has been linked to elevated depression, anxiety, and stress (3), which in turn can impair clinical performance, increase medical errors, and diminish patient safety (4–6). The pandemic further intensified these vulnerabilities, and multiple studies have demonstrated sustained psychological consequences (7–9).

Emerging evidence from the Gulf region suggests that the mental health burden among HCWs may be even greater than global averages, with more than half of providers screening positive for depression and reporting elevated levels of stress and anxiety (10). These findings raise concern for the long−term well−being of the regional healthcare workforce, especially in the context of ongoing workforce shortages, rapid health−system expansion, and high service demand in Gulf Cooperation Council (GCC) countries. In the United Arab Emirates (UAE), however, evidence on the post−pandemic mental health status of HCWs remains limited. Most available studies were conducted during the acute pandemic phase and focused on frontline HCWs’ psychological symptoms, occupational risks, and experiences of work−related stress (11–14). Little is known about mental health outcomes among HCWs in the post−pandemic period, nor about the demographic, occupational, and medical determinants of mental distress in the UAE context.

Understanding HCWs’ mental health outcomes and their determinants in this setting is essential for workforce resilience and health−system preparedness (15). Poor psychological well−being among HCWs has been associated with absenteeism, reduced productivity, lower job satisfaction, and higher turnover, outcomes that can compromise care delivery and emergency response capacity (16–18). These risks are particularly salient in health systems that rely heavily on expatriate clinicians and aim to sustain high−quality services while managing rising demand.

Informed by prior literature applying multilevel and socio-ecological frameworks to occupational mental health (19–23), we conceptualised HCW mental health as the outcome of interacting demographic characteristics, occupational exposures, medical conditions, and organizational factors, which together influence depression, anxiety, and stress. Ongoing assessment of factors associated with HCWs’ mental health distress is therefore crucial to guide occupational health policies, targeted interventions, and support programs tailored to high−risk groups. This cross−sectional study was conducted among physicians and nurses providing direct clinical care within Abu Dhabi Health Services Company (SEHA), the main public healthcare provider in the emirate of Abu Dhabi. The study had two main objectives: first, to estimate the prevalence and severity of depression, anxiety, and stress among frontline doctors and nurses in Abu Dhabi using the validated Depression, Anxiety, and Stress Scale (DASS−21); and second, to examine demographic, occupational, and medical determinants associated with these mental health outcomes.

## 2 Materials and methods

### 2.1 Study area, design, and population

We conducted a cross−sectional electronic survey to estimate the prevalence and determinants of depression, anxiety, and stress among HCWs in Abu Dhabi, United Arab Emirates. The study was carried out within SEHA, the main public healthcare provider in the emirate, which operates 13 public hospitals and 36 primary care facilities and employs approximately 3,841 physicians and 2,925 nurses providing direct clinical care. Abu Dhabi is the largest emirate in the UAE and hosts a multi−national healthcare workforce serving a rapidly growing population, making it a critical setting for understanding post−pandemic mental health among HCWs.

The target population comprised physicians (residents, specialists, consultants) and nurses involved in direct patient care across SEHA−affiliated hospitals and primary health centres. The optimal sample size of 364 participants was calculated using the Leslie–Kish formula for single−proportion estimation and the Raosoft sample−size calculator, based on the highest locally reported prevalence of anxiety (51.5%) among SEHA providers (11), a 95% confidence interval, a 5% margin of error, and a finite population of 6,769 eligible HCWs.

### 2.2 Data collection and measures

With administrative permission, official SEHA email lists were used to distribute the survey link to eligible HCWs. Invitation emails included brief study information, an electronic informed−consent statement, and a secure link to the questionnaire (Google Forms). Participation was voluntary and anonymous, and no incentives were offered. Recruitment was supported by reminder emails and internal communication announcements. Data collection took place in March 2024.

The survey captured sociodemographic characteristics (age, gender, marital status, education, income, nationality, race, residence, living situation). Occupational history and work characteristics (job type, years of experience, workplace setting, shift length, work duration, medication errors, needlestick/sharps injuries, conflicts with colleagues, conflicts with patients or family members, communication difficulties with patients, and COVID−19–related changes in working hours or job responsibilities). Self−reported medical history (mental health conditions, hypertension, diabetes, thyroid disease, chronic neck pain, low back pain, chronic fatigue, and COVID−19 infection in the last two years).

Mental health outcomes were measured using DASS−21. Each subscale (7 items) was scored on a 0–3 Likert scale, summed, and multiplied by two to yield final subscale scores, which were then categorized according to standard severity thresholds. The survey instrument and online flow were piloted among 20 HCWs to assess clarity, comprehension, and technical functionality; pilot responses were excluded from the main analysis.

### 2.3 Statistical analysis

Data were analysed using SPSS version 28. Participant characteristics and DASS−21 scores were summarised using frequencies and percentages for categorical variables and medians with interquartile ranges for continuous variables. Associations between mental health outcomes (presence of depression, anxiety, and stress based on DASS−21 cut−offs) and covariates were examined using Pearson’s χ² test or Fisher’s exact test where appropriate. Variables with p<0.20 in bivariate analyses were entered into multivariable logistic regression to be conservative and reduce the risk of excluding potentially important predictors. We estimated the crude and adjusted odds ratios (aORs) and 95% confidence intervals for each outcome. Statistical significance was set at p<0.05.

### 2.4 Ethical consideration

The study was conducted in accordance with the ethical principles of the Declaration of Helsinki. Ethical approvals were obtained from the United Arab Emirates University Social Sciences Ethics Committee (IRB Approval No: ERSC2022−1806) and the SEHA Research Ethics Committee (IRB Approval No: HREC SEHA−IRB−256). Electronic informed consent was required before survey initiation. No identifiable data were collected, and all responses were anonymous and handled with strict confidentiality.

## 3 Results

### 3.1 Participant characteristics

A total of 383 healthcare workers (HCWs) were included in the study, with a mean age of 40.8 years (SD = 8.7). The majority were female (70%), married (74%), and nurses (65%). Most respondents held at least a bachelor’s degree (69%), with 22% reporting postgraduate qualifications. In terms of residence, nearly seven in ten participants lived in Abu Dhabi, and 74% resided with their families (**Supplementary Table 1**). Occupationally, two-thirds of HCWs were nurses, and almost three-quarters were employed in hospital settings. More than one-third had less than five years of work experience, and about 69% reported a change in working hours during the COVID-19 pandemic (**Supplementary Table 2**). Regarding medical history, low back pain (31%) and hypertension (15%) were the most common chronic conditions, while only 2.6% reported a diagnosed mental health condition (**Supplementary Table 3**).

### 3.2 Prevalence of depression, anxiety, and stress

**Figure 1:**
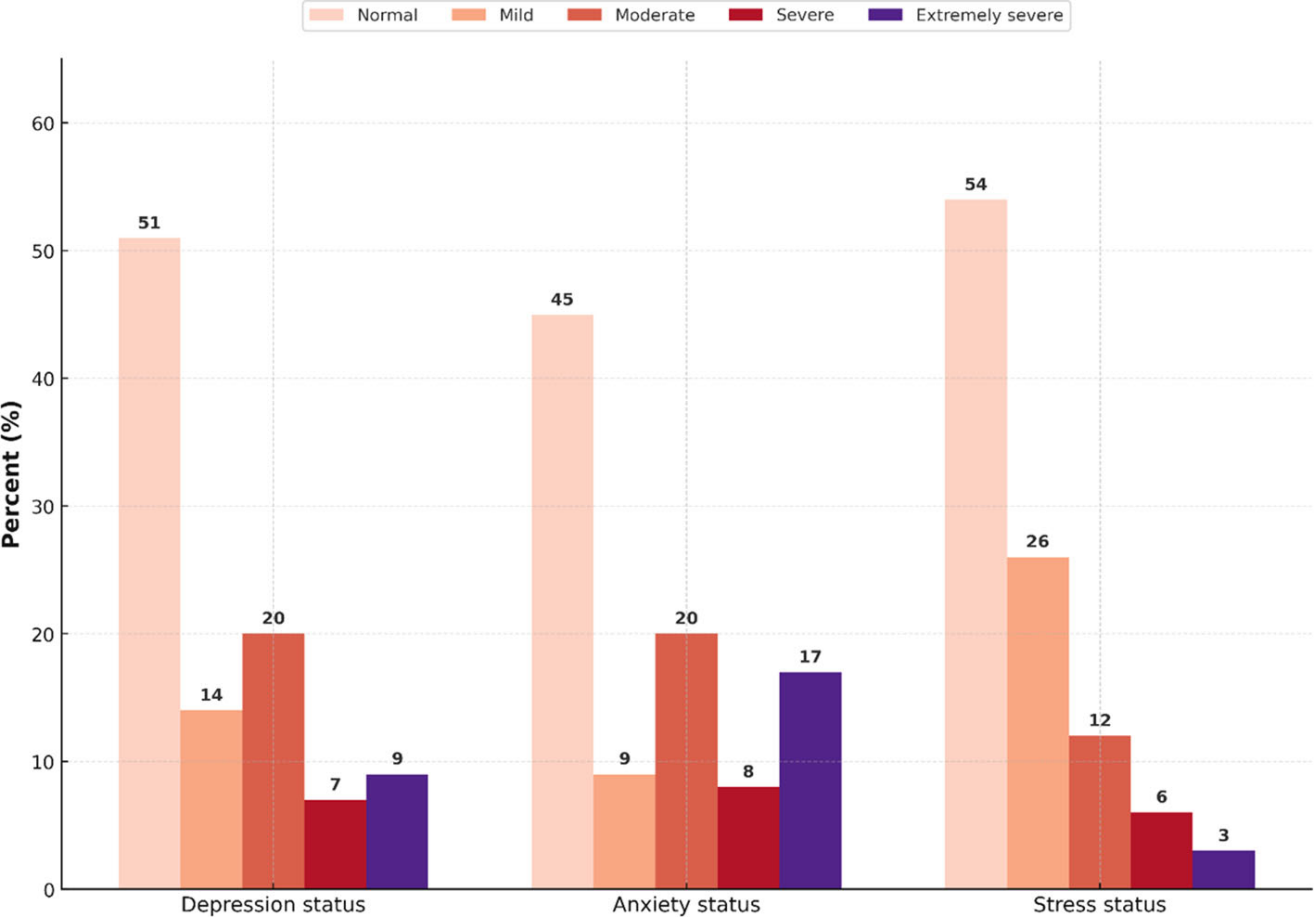
Depression, anxiety, and stress status of the participants (N = 383).

The median scores (IQR) were 8.0 (2.0–16.0) for both depression and anxiety, and 10.0 (4.0–16.0) for stress. Using the DASS-21 cut-offs, the prevalence of depression, anxiety, and stress among HCWs was 49%, 45%, and 46%, respectively (**Figure 1**).

### 3.3 Determinants of mental health distress

#### 3.3.1 Demographic and occupational factors

Multivariable logistic regression revealed that HCWs of Middle Eastern origin were significantly more likely to report depression (aOR = 1.94, 95% CI: 1.13–3.35), anxiety (aOR = 1.80, 95% CI: 1.03–3.21), and stress (aOR = 2.42, 95% CI: 1.37–4.33), compared to Asian HCWs (**Table 1**). HCWs aged ≥40 years had lower odds of stress (aOR = 0.61, 95% CI: 0.39–0.96), while those with postgraduate qualifications were less likely to experience anxiety (aOR = 0.55, 95% CI: 0.31–0.94).

**Table 1:** Demographic factors associated with depression, anxiety, and stress.

Characteristics	Depression		Anxiety		Stress	
	cOR (95% CI)	aOR (95% CI)	cOR (95% CI)	aOR (95% CI)	cOR (95% CI)	aOR (95% CI)
Age						
< 40	Ref	Ref	Ref	Ref	Ref	Ref
≥ 40	0.85 (0.56, 1.27)	—	**0.58 (0.38, 0.87)**	0.68 (0.44, 1.05)	**0.62 (0.41, 0.93)**	**0.61 (0.39, 0.96)**
Gender						
Female	Ref	Ref	Ref	Ref	Ref	Ref
Male	0.83 (0.54, 1.29)	—	0.73 (0.47, 1.13)	0.74 (0.46, 1.18)	1.07 (0.69, 1.66)	—
Education						
Diploma or bachelor	Ref	Ref	Ref	Ref	Ref	Ref
Postgraduate	1.22 (0.78, 1.92)	—	0.65 (0.41, 1.01)	**0.55 (0.31, 0.94)**	1.2 (0.76, 1.89)	—
Income (AED)						
≤ 20,000	Ref	Ref	Ref	Ref	Ref	Ref
> 20,000	0.95 (0.63, 1.43)	—	0.77 (0.51, 1.15)	0.7 (0.43, 1.13)	1.24 (0.82, 1.86)	—
Profession						
Nurse	Ref	Ref	Ref	Ref	Ref	Ref
Physician	0.64 (0.36, 1.12)	—	1.09 (0.62, 1.91)	—	**0.41 (0.23, 0.73)**	0.59 (0.27, 1.26)
Others	0.65 (0.32, 1.28)	—	0.7 (0.35, 1.38)	—	**0.39 (0.19, 0.78)**	0.5 (0.22, 1.13)
Marital status						
Married	Ref	Ref	Ref	Ref	Ref	Ref
Unmarried	**1.60 (1.01, 2.54)**	1.71 (0.92, 3.24)	1.24 (0.78, 1.97)	—	1.41 (0.89, 2.22)	1.13 (0.59, 2.17)
Nationality						
Emiratis	Ref	Ref	Ref	Ref	Ref	Ref
Non-Emiratis	0.65 (0.34, 1.22)	1.16 (0.52, 2.56)	0.68 (0.34, 1.29)	0.64 (0.27, 1.51)	**0.41 (0.21, 0.79)**	0.98 (0.38, 2.51)
Race						
Asian	Ref	Ref	Ref	Ref	Ref	Ref
African	2.00 (0.80, 5.27)	1.85 (0.72, 5.02)	1.75 (0.69, 4.80)	2.33 (0.84, 6.98)	2.05 (0.82, 5.28)	1.96 (0.73, 5.32)
Middle eastern	**2.03 (1.26, 3.31)**	**1.94 (1.13, 3.35)**	1.57 (0.97, 2.57)	**1.8 (1.03, 3.21)**	**2.8 (1.72, 4.60)**	**2.42 (1.37, 4.33)**
Caucasian	1.33 (0.55, 3.23)	1.32 (0.53, 3.30)	0.94 (0.39, 2.28)	1.4 (0.54, 3.67)	2.01 (0.83, 4.96)	1.89 (0.75, 4.85)
Residence						
Abu Dhabi	Ref	Ref	Ref	Ref	Ref	Ref
Al Ain	1.36 (0.88, 2.13)	1.37 (0.85, 2.20)	1.03 (0.66, 1.61)	—	1.43 (0.92, 2.24)	1.23 (0.75, 2.02)
Others	3.49 (0.44, 71.0)	2.05 (0.17, 47.3)	2.54 (0.32, 51.7)	—	3.94 (0.50, 80.2)	1.31 (0.08, 38.6)
Living situation						
Alone	Ref	Ref	Ref	Ref	Ref	Ref
Shared accommodation	0.65 (0.21, 2.00)	0.89 (0.27, 2.83)	1.6 (0.52, 5.36)	—	0.40 (0.12, 1.24)	0.64 (0.18, 2.13)
With family	0.67 (0.35, 1.26)	1.06 (0.46, 2.43)	1.05 (0.56, 1.98)	—	0.58 (0.30, 1.10)	0.67 (0.28, 1.59)
With partner	0.58 (0.24, 1.41)	1.03 (0.38, 2.83)	0.88 (0.36, 2.11)	—	0.73 (0.30, 1.76)	0.78 (0.27, 2.22)

cOR, Crude Odds Ratio; aOR, Adjusted Odds Ratio; CI, Confidence Interval; Ref, Reference category. Bold : p<0.05.

Occupationally, nurses had significantly lower odds of stress compared to doctors (aOR = 0.47, 95% CI: 0.23–0.94). Duration of work also contributed to mental health, as those with 11–15 years of service were less likely to experience stress than those with ≤5 years (aOR = 0.51, 95% CI: 0.26–1.00) (**Table 2**).

**Table 2:** Occupational factors associated with depression, anxiety, and stress.

Characteristics	Depression		Anxiety		Stress	
	cOR (95% CI)	aOR (95% CI)	cOR (95% CI)	aOR (95% CI)	cOR (95% CI)	aOR (95% CI)
Job type						
Doctor	Ref	Ref	Ref	Ref	Ref	Ref
Nurse	0.64 (0.36, 1.12)	0.65 (0.35, 1.21)	1.09 (0.62, 1.91)	—	**0.41 (0.23, 0.73)**	**0.47 (0.23, 0.94)**
Others	0.65 (0.32, 1.28)	0.7 (0.33, 1.47)	0.7 (0.35, 1.38)	—	**0.39 (0.19, 0.78)**	0.49 (0.22, 1.11)
Workplace						
Hospital	Ref	Ref	Ref	Ref	Ref	Ref
Clinic	0.77 (0.45, 1.31)	—	0.91 (0.54, 1.56)	—	0.61 (0.35, 1.05)	0.65 (0.35, 1.19)
Both	0.82 (0.40, 1.67)	—	0.96 (0.48, 1.97)	—	1.67 (0.82, 3.49)	1.22 (0.51, 2.89)
Work duration						
0-5	Ref	Ref	Ref	Ref	Ref	Ref
6-10	0.91 (0.53, 1.54)	—	0.72 (0.42, 1.22)	0.84 (0.48, 1.46)	0.67 (0.39, 1.14)	0.83 (0.45, 1.49)
11-15	0.82 (0.46, 1.46)	—	0.57 (0.32, 1.00)	0.68 (0.37, 1.25)	**0.38 (0.20, 0.68)**	0.51 (0.26, 1.00)
> 15	1.47 (0.84, 2.58)	—	1.34 (0.76, 2.38)	1.57 (0.87, 2.88)	0.82 (0.47, 1.42)	0.95 (0.52, 1.76)
Medication error						
No	Ref	Ref	Ref	Ref	Ref	Ref
Yes	**2.35 (1.02, 5.89)**	1.26 (0.47, 3.57)	0.9 (0.40, 2.04)	—	**4.01 (1.65, 11.2)**	1.84 (0.63, 5.94)
Needle stick/sharps injury						
No	Ref	Ref	Ref	Ref	Ref	
Yes	1.79 (0.83, 4.02)	1.06 (0.42, 2.70)	1.64 (0.76, 3.77)	1.13 (0.48, 2.79)	2.01 (0.93, 4.50)	0.73 (0.27, 1.95)
Conflict with colleagues						
No	Ref	Ref	Ref	Ref	Ref	Ref
Yes	**3.94 (2.31, 6.93)**	**3.36 (1.92, 6.05)**	**2.52 (1.50, 4.37)**	**1.99 (1.14, 3.53)**	**4.89 (2.85, 8.70)**	**3.78 (2.10, 7.00)**
Conflicts with patients and family members						
No	Ref	Ref	Ref	Ref	Ref	
Yes	**2.53 (1.48, 4.42)**	1.63 (0.88, 3.04)	**2.42 (1.40, 4.34)**	**1.87 (1.02, 3.54)**	**3.37 (1.95, 6.00)**	**2.12 (1.11, 4.14)**
Communication difficulties with patients						
Daily	Ref	Ref	Ref	Ref	Ref	Ref
Not daily	**0.62 (0.38, 0.99)**	**0.59 (0.36, 0.98)**	**0.53 (0.32, 0.86)**	**0.55 (0.33, 0.91)**	**0.54 (0.33, 0.86)**	**0.48 (0.29, 0.81)**
Never	**0.47 (0.24, 0.94)**	0.58 (0.27, 1.19)	**0.32 (0.16, 0.65)**	**0.39 (0.19, 0.80)**	**0.29 (0.14, 0.59)**	**0.38 (0.17, 0.83)**
Work our change during COVID-19 pandemic						
No	Ref	Ref	Ref	Ref	Ref	Ref
Yes	1.11 (0.72, 1.71)	—	1.05 (0.68, 1.62)	—	0.91 (0.59, 1.40)	—
Change of job responsibility/location						
No	Ref	Ref	Ref	Ref	Ref	Ref
Yes	1.38 (0.86, 2.24)	1.25 (0.75, 2.07)	1.47 (0.91, 2.40)	1.29 (0.77, 2.17)	1.26 (0.78, 2.02)	—

cOR, Crude Odds Ratio; aOR, Adjusted Odds Ratio; CI, Confidence Interval; Ref, Reference category. Bold : p<0.05.

Conflict at the workplace emerged as a critical determinant. HCWs who reported conflicts with colleagues had significantly higher odds of depression (aOR = 3.36, 95% CI: 1.92–6.05), anxiety (aOR = 1.99, 95% CI: 1.14–3.53), and stress (aOR = 3.78, 95% CI: 2.10–7.00). Similarly, conflicts with patients or family members were associated with higher anxiety (aOR = 1.87, 95% CI: 1.02–3.54) and stress (aOR = 2.12, 95% CI: 1.11–4.14). Interestingly, HCWs who reported no daily communication difficulties with patients showed significantly lower odds of depression, anxiety, and stress (**Table 2**).

#### 3.3.2 Medical factors

Medical conditions also influenced mental health outcomes. HCWs with pre-existing mental health conditions had substantially higher odds of stress (aOR = 7.37, 95% CI: 1.27–139). Chronic fatigue was strongly associated with depression (aOR = 4.21, 95% CI: 1.61–13.2), anxiety (aOR = 3.89, 95% CI: 1.39–13.8), and stress (aOR = 3.34, 95% CI: 1.32–9.63). Additionally, low back pain was significantly associated with higher odds of depression and stress, though associations were attenuated after adjustment. COVID-19 infection history did not significantly influence the odds of any of the three outcomes (**Table 3**).

**Table 3:** Medical factors associated with depression, anxiety, and stress.

Characteristics	Depression		Anxiety		Stress	
	cOR (95% CI)	aOR (95% CI)	cOR (95% CI)	aOR (95% CI)	cOR (95% CI)	aOR (95% CI)
Mental health conditions						
No	Ref	Ref	Ref	Ref	Ref	Ref
Yes	**4.34 (1.07, 29.0)**	2.66 (0.59, 18.6)	**7.78 (1.44, 144)**	4.94 (0.84, 93.7)	**11.0 (2.03, 204)**	**7.37 (1.27, 139)**
Diabetes type 2						
No	Ref	Ref	Ref	Ref		
Yes	0.55 (0.25, 1.16)	0.6 (0.26, 1.28)	0.76 (0.36, 1.60)	—	0.62 (0.28, 1.30)	0.68 (0.30, 1.47)
Hypertension						
No	Ref	Ref	Ref	Ref	Ref	Ref
Yes	1.02 (0.58, 1.77)	—	0.91 (0.52, 1.59)	—	1.06 (0.61, 1.85)	—
Thyroid disease						
No	Ref		Ref	Ref	Ref	Ref
Yes	0.77 (0.35, 1.67)	—	0.51 (0.23, 1.11)	0.52 (0.23, 1.16)	0.63 (0.27, 1.37)	—
Chronic neck pain						
No	Ref	Ref	Ref	Ref	Ref	Ref
Yes	**2.19 (1.13, 4.44)**	1.31 (0.62, 2.85)	**2.48 (1.24, 5.33)**	1.75 (0.80, 4.04)	**2.47 (1.27, 5.00)**	1.49 (0.70, 3.24)
Low back pain						
No	Ref	Ref	Ref	Ref	Ref	Ref
Yes	**1.63 (1.05, 2.54)**	1.35 (0.85, 2.18)	1.43 (0.92, 2.24)	1.15 (0.72, 1.87)	**1.63 (1.06, 2.54)**	1.37 (0.85, 2.20)
Chronic fatigue						
No	Ref	Ref	Ref	Ref	Ref	Ref
Yes	**5.36 (2.15, 16.2)**	**4.21 (1.61, 13.2)**	**5.51 (2.08, 19.0)**	**3.89 (1.39, 13.8)**	**4.73 (1.99, 13.1)**	**3.34 (1.32, 9.63)**
COVID-19 in the last 2 years						
No	Ref	Ref	Ref	Ref	Ref	Ref
Yes	1.3 (0.81, 2.10)	—	1.1 (0.68, 1.77)	1.05 (0.64, 1.72)	1.36 (0.84, 2.21)	1.34 (0.82, 2.22)

cOR, Crude Odds Ratio; aOR, Adjusted Odds Ratio; CI, Confidence Interval; Ref, Reference category. Bold : p<0.05.

## 4 Discussion

This study provides one of the most recent assessments of depression, anxiety, and stress among HCWs in Abu Dhabi during the post−pandemic phase. We found a substantial mental health burden, with nearly half of participants reporting clinically significant anxiety and approximately half screening positive for depression and stress. These rates mirror earlier Abu Dhabi data collected during the height of the pandemic and suggest that psychological distress has remained elevated even after acute transmission pressures have eased. The persistence of symptoms supports the view that pandemic−related strain has evolved into a prolonged occupational stressor rather than a short−term crisis response.

The prevalence estimates in this study fall within the higher range of recent international reports. Meta−analyses conducted during COVID−19 commonly report that one−quarter to one−third of HCWs globally experienced depression or anxiety, with higher figures in frontline settings (7–9). Our findings are also similar to those reported from Saudi Arabia, Qatar, and Egypt, where 40–69% of HCWs screened positive for moderate−to−severe symptoms (10, 24, 25). The similarity between our results and broader GCC trends suggests shared occupational pressures, workforce composition, and sociocultural factors, including stigma surrounding help−seeking, rapidly expanding service demand, and heavy reliance on expatriate clinicians (26, 27).

Several demographic and professional characteristics were associated with increased mental distress. Younger HCWs consistently reported higher depression, anxiety, and stress, in line with earlier data from SEHA staff during the pandemic and international literature on early−career clinicians (11). Younger doctors and nurses typically have less experience, more limited coping resources, greater job insecurity, and higher exposure to emotionally taxing duties, which may heighten vulnerability to distress (28–31). In our study, medical residents and junior nurses exhibited the highest levels of severe symptoms, in line with global evidence that trainees represent one of the most vulnerable professional groups, particularly when redeployed to high−acuity settings during and after health emergencies (32–36).

Occupational role and workplace environment also influenced mental health outcomes. Providers working in emergency, critical care, and obstetrics and gynaecology reported higher symptom scores compared with those in outpatient or primary care settings. These high−intensity specialties manage unpredictable emergencies, rapid clinical deterioration, and ethically complex decisions, stressors that were amplified during COVID−19 surges and may have persisted as services normalized. Our findings are consistent with international evidence that psychological risk is not evenly distributed across the healthcare workforce but clusters in frontline units with frequent exposure to critical illness, trauma, and patient mortality (37).

We observed notable differences by race and medical history. Middle Eastern HCWs had significantly higher odds of depression, anxiety, and stress compared with Asian HCWs, which may reflect differences in coping strategies, social support networks, financial responsibilities, or perceived job security. Chronic fatigue, pre−existing mental health conditions, and musculoskeletal pain, particularly low back pain, were strongly associated with adverse mental health outcomes. These findings highlight the interplay between physical and psychological health and underscore the importance of integrated occupational health approaches that address both domains.

Interestingly, we did not detect significant gender differences in depression or anxiety, which contrasts with global findings of higher distress among female HCWs (38–44), but aligns with previous studies from the UAE (11, 12). Cultural and workforce characteristics may partly explain this pattern. The UAE healthcare system relies heavily on expatriate staff, and both male and female providers often lack an extended family support network, potentially equalizing stress exposure across genders (45). However, self−report measures may also mask gendered differences in symptom disclosure, and further research is needed to explore how gender norms and cultural expectations shape help−seeking and reporting among HCWs in the region.

Our findings have important implications for policy and practice. Short−term interventions introduced during the pandemic, such as mental health hotlines and *ad hoc* counselling services, were valuable but are unlikely to be sufficient for long−term recovery. Healthcare organizations should institutionalize structured and confidential employee assistance programmes, routine mental−health screening, and clear referral pathways for staff in distress, with particular attention to younger professionals, trainees, and those in high−intensity specialties. Policies that reduce administrative overload, optimize shift scheduling, and protect rest periods could help address fatigue and sleep disturbance, which are strong predictors of burnout and depression. In addition, conflict−resolution and communication training, along with interventions to improve team climate and reduce workplace conflict, may mitigate key occupational risk factors identified in this study.

At the system level, embedding mental−health and well−being indicators into workforce planning, quality−of−care monitoring, and accreditation standards could support sustained attention to HCW mental health. Tailored programmes such as mentorship schemes for early−career staff, psychological debriefing for high−acuity units, and resilience training integrated into residency curricula may further strengthen coping capacity and retention.

This study has several strengths. It provides timely, post−pandemic evidence from the largest public health network in Abu Dhabi, uses a validated assessment tool (DASS−21), and applies multivariable logistic regression to examine a broad set of demographics, occupational, and medical determinants. Nonetheless, important limitations should be acknowledged. The cross−sectional design precludes causal inference, and self−reported measures may under− or overestimate the true prevalence of depression, anxiety, and stress. Participation was voluntary, which may introduce response bias if individuals with higher or lower distress were differentially likely to respond. In addition, the study focused on a single emirate and provider network, which may limit generalisability to other settings in the UAE. We also did not assess other relevant mental health outcomes, such as post−traumatic stress disorder, sleep disorders, or burnout, to avoid excessive respondent burden. Future longitudinal studies and mixed−methods research are needed to track changes in HCW mental health over time, explore underlying mechanisms in greater depth, and evaluate the impact of specific organizational and policy interventions.

## 5 Conclusion

Healthcare workers in Abu Dhabi continue to experience substantial psychological distress in the post-pandemic period, with younger staff, trainees, and high-intensity specialties at greatest risk. Protecting the mental health of healthcare providers is essential not only for their well-being, but for patient care quality and system sustainability. There is an urgent need for sustained, organizationally supported mental-health programs, routine screening, and targeted interventions for high-risk groups. By embedding mental-health support into workforce policy and clinical practice, the UAE can strengthen the resilience of its healthcare system and set a regional benchmark for safeguarding the well-being of its clinical workforce.

## Data Availability

The datasets presented in this article are not readily available because of institutional data protection regulations and SEHA data-sharing policies. As per SEHA Research Ethics Committee requirements, raw patient-level data may only be shared under controlled access to protect confidentiality and comply with ethical and legal standards. Researchers who meet the institutional criteria for access to confidential data may request the dataset from the corresponding author, subject to approval by the SEHA Research Ethics Committee and completion of a formal data-sharing agreement. Requests to access the datasets should be directed to ielbarazi@uaeu.ac.ae.
