# Supplementary material for: Determinants of mental health distress among health workers in Abu Dhabi, United Arab Emirates
Source: Front Psychiatry. 2026 Jun 2;17:1756380. doi: 10.3389/fpsyt.2026.1756380 (PMC13269354; doi:10.3389/fpsyt.2026.1756380)
Supplement: Supplementary file 1 [file Table1.docx]

**Supplementary Tables**

**Table S1: Participant's demographic characteristics (N=383)**

| Demographic characteristics | | Frequency (%) |
| --- | --- | --- |
|  | **Age, mean (SD)** | 40.8 (8.7) |
| Gender | Female | 267 (70%) |
|  | Male | 116 (30%) |
| Marital status | Married | 282 (74%) |
|  | Unmarried | 101 (26%) |
| Education | Diploma | 19 (5.0%) |
|  | Post graduate diploma | 13 (3.4%) |
|  | Bachelor | 261 (69%) |
|  | Masters | 69 (18%) |
|  | PhD or equivalent | 18 (4.7%) |
| Income (AED) | 5,000 - 10,000 | 52 (14%) |
|  | 11,000 - 15,000 | 156 (41%) |
|  | 16,000 - 20,000 | 71 (19%) |
|  | 21,000 - 25,000 | 19 (5.0%) |
|  | 26,000 - 30,000 | 14 (3.7%) |
|  | More than 30,000 | 67 (18%) |
|  |  |  |
| Nationality | Emiratis | 43 (11%) |
|  | Non-Emiratis | 338 (89%) |
| Race | Asian | 233 (63%) |
|  | African | 20 (5.4%) |
|  | Middle eastern | 96 (26%) |
|  | Caucasian | 22 (5.9%) |
| Residence | Abu Dhabi | 266 (69%) |
|  | Al Ain | 113 (30%) |
|  | Others | 4 (1.0%) |
| Living situation | Alone | 45 (12%) |
|  | Shared accommodation | 17 (4.5%) |
|  | With family | 284 (74%) |
|  | With partner | 36 (9.4%) |
| Number of persons living with | One | 71 (19%) |
|  | Two | 66 (17%) |
|  | Three | 72 (19%) |
|  | Four | 66 (17%) |
|  | Five | 40 (10%) |
|  | More than five | 68 (18%) |

**Table S2: Participant's occupational characteristics (N=383)**

| **Characteristics** | **Frequency (%)** |
| --- | --- |
| **Job type** |  |
| Doctor | 62 (16%) |
| Nurse | 249 (65%) |
| Others | 72 (19%) |
| **Workplace** |  |
| Hospital | 277 (73%) |
| Clinic | 68 (18%) |
| Both | 35 (9.2%) |
| **Work duration** |  |
| 0-5 | 151 (39%) |
| 6-10 | 87 (23%) |
| 11-15 | 69 (18%) |
| > 15 | 76 (20%) |
| **Medication error** |  |
| No | 358 (93%) |
| Yes | 25 (6.5%) |
| **Needle stick/ sharps injury** |  |
| No | 354 (92%) |
| Yes | 29 (8%) |
| **Conflict with colleagues** |  |
| No | 302 (79%) |
| Yes | 81 (21%) |
| **Conflicts with patients and family members** |  |
| No | 313 (82%) |
| Yes | 70 (18%) |
| **Communication difficulties with patients** |  |
| Daily | 101 (26%) |
| Not daily | 232 (61%) |
| Never | 50 (13%) |
| **Work our change during COVID-19pandemic** |  |
| No | 119 (31%) |
| Yes | 264 (69%) |
| **Change of job responsibility/work location** |  |
| No | 294 (77%) |
| Yes | 89 (23%) |

**Table S3: Participant's medical history (N=383)**

| **Characteristics** | **Frequency (%)** |
| --- | --- |
| **Mental health conditions** |  |
| No | 373 (97%) |
| Yes | 10 (2.6%) |
| **Diabetes type 2** |  |
| No | 352 (92%) |
| Yes | 31 (8.1%) |
| **Hypertension** |  |
| No | 324 (85%) |
| Yes | 59 (15%) |
| **Thyroid disease** |  |
| No | 355 (93%) |
| Yes | 28 (7.3%) |
| **Chronic neck pain** |  |
| No | 342 (89%) |
| Yes | 41 (11%) |
| **Low back pain** |  |
| No | 266 (69%) |
| Yes | 117 (31%) |
| **Chronic fatigue** |  |
| No | 355 (93%) |
| Yes | 28 (7.3%) |
| **COVID-19 in the last 2 years** |  |
| No | 89 (23%) |
| Yes | 290 (77%) |
